# Longitudinal proxy measurements in multiple sclerosis: patient-proxy agreement on the impact of MS on daily life over a period of two years

**DOI:** 10.1186/1471-2377-8-2

**Published:** 2008-02-28

**Authors:** Femke AH van der Linden, Jolijn J Kragt, Margarethe van Bon, Martin Klein, Alan J Thompson, Henk M van der Ploeg, Chris H Polman, Bernard MJ Uitdehaag

**Affiliations:** 1 Department of Neurology, VU University Medical Centre, Amsterdam, The Netherlands; 2 Medical Psychology, VU University Medical Centre, Amsterdam, The Netherlands; 3 Clinical Epidemiology and Biostatistics, VU University Medical Centre, Amsterdam, The Netherlands; 4 Neurological Outcome Measures Unit, Institute of Neurology, London, UK

## Abstract

**Background:**

The use of self-report measurements in clinical settings is increasing. However, in patients with limitations that interfere with reliable self-assessment such as cognitive impairment or mood disturbances, as may be the case in multiple sclerosis (MS), data collection might be problematic. In these situations, information obtained from proxy respondents (e.g. partners) may replace self-ratings. The aim of this study was to examine the value of proxy ratings at separate points in time and to assess patient-proxy agreement on possible changes in disease impact of MS.

**Methods:**

Fifty-six MS patients and their partners completed the Multiple Sclerosis Impact Scale (MSIS-29) at baseline and follow-up, two years later. Patient-proxy agreement was assessed at both time points by calculating intraclass correlation coefficients (ICCs), exact and global agreement and the mean directional differences between groups. Agreement of change over time was assessed by calculating ICCs between change scores. In parallel, global ratings of both patients and proxy respondents of the extent to which the patient had improved or deteriorated over the past two years were collected to validate possible changes on the MSIS-29.

**Results:**

At both time points, agreement on the physical scale was higher than agreement on the psychological scale (ICCs at baseline were 0.81 for the physical scale and 0.72 for the psychological scale; at follow-up, the ICC values were 0.86 and 0.65 respectively). At follow-up, statistically significant mean differences between patients and proxies were noted for the physical scale (-4.8 ± 12.7, p = 0.006) and the psychological scale (-8.9 ± 18.8, p = 0.001). Agreement between change scores on the MSIS-29 was fair (ICC < 0.60). Our analyses suggest that the validity of measuring changes over time might be better for proxy respondents compared to patients.

**Conclusion:**

Proxy respondents could act as a reliable source of information in cross-sectional studies. Moreover, results suggested that agreement on change over time might be better for proxy respondents compared to patients. Although this remarkable finding should be interpreted cautiously because of several limitations of the study, it does plead for further investigation of this important topic.

## Background

Outcomes of health status measurements are becoming increasingly important in evaluating the treatment effect of clinical trials and could potentially play an important role in medical decision-making. Health status measurements can be used to measure cross-sectional differences in health status between patients at a certain point in time or longitudinal changes in health status within patients over a period of time [1]. Data collection is mainly performed by using self-report questionnaires to capture the patient's perspective. In recent years, several self-report measures have been developed to assess impairment and disability of Multiple Sclerosis (MS). Of those available, the Multiple Sclerosis Impact Scale (MSIS-29) is both disease specific and rigorously evaluated for its psychometric properties [2-4]. The MSIS-29 measures the disease impact of MS on daily life of MS patients. Although it is generally agreed that the patient is the best rater of their own health status, conditions such as cognitive impairment or mood disturbance might lead to inaccurate self-report or even loss of information due to missing data. This could result in data which are not representative for the patient population of interest. Exclusion of such patients, a sometimes chosen approach, may cause bias in the assessment of health status.

In MS, cognitive impairment is an important issue [5]. Research has shown that cognitive deficits are present in 40 to 60 percent of MS patients [5,6]. During the different disease stages and subtypes of MS, variable cognitive profiles are seen and disturbances may start already early in the disease, even before serious physical disability has developed [5,7]. Mood disturbances (depression and anxiety) may also occur during the disease course of MS. Depression is common in approximately half of the MS patients [8]. Anxiety disorders present themselves in about 36% of the MS patients [9]. The frequent use of self-report measurements and the common presence of cognitive impairment and mood disturbances could cause a problem in MS research. A possible solution for this problem might be the incorporation of a third person, a so-called proxy respondent who can provide information on the health status of the patient that otherwise would be inaccurate or even lost [10]. The use of proxy respondents is based on the assumption that a proxy respondent is capable of assessing the values and preferences of the patient. Besides this, the proxy respondent should be able to identify any changes that might occur over time in the patient's view [11]. So far, little is known about the value of proxy respondents in MS. A previously performed cross-sectional study on patient-proxy agreement in a small MS sample indicated that partners might be useful sources of information when assessing the impact of MS on daily life of patients [12]. Although this is an important finding, the stability of these findings over time remains to be investigated. The validity of measuring changes over time in a longitudinal setting, for example in clinical trials in which the effect of treatment is assessed over time, is especially important. Several studies have examined patient-proxy agreement on change over time in a longitudinal setting, but diverse results were reported [13-19]. Sneeuw et al. provided support for the responsiveness to change of significant others and health care providers [15,18]. A few studies indicated an improvement of patient-proxy agreement over time when patients improved or became stable over time [13,14,19]. Yet, other studies reported contrary results by showing poor patient-proxy agreement on changes over time [16,17].

Until now, no longitudinal studies on patient-proxy agreement were performed in MS.

The objective of this study was to examine patient-proxy agreement on the disease impact of MS on daily life at two separate points in time, and to assess patient-proxy agreement on possible changes between those two time points.

## Methods

### Study design

This prospective longitudinal cohort study was conducted at the MS Center of the VU University Medical Center (Amsterdam, The Netherlands). Data was collected at baseline and two years after baseline. The medical ethical committee of the VU University Medical Center approved the study protocol and informed consent was obtained from all participants prior to participation.

### Measures and procedures

#### Baseline

At baseline, patients and proxy respondents were recruited from an ongoing study at the MS Center. This was a case-control study in Dutch MS patients and matched healthy controls to examine whether serum vitamin D levels are associated with the risk of MS. Inclusion criteria for both patients and healthy controls were: written informed consent and age between 18 and 75 years. Exclusion criteria included (medical) conditions that were thought to highly influence the serum concentrations of vitamin D metabolites. In addition, healthy controls should have a negative family history regarding MS. In case the healthy control person was the partner of the patient, he or she was asked to participate as a proxy respondent in the present study.

Both patients and proxy respondents were asked to complete the MSIS-29 [see additional file 1]. This was done independently from each other in separate rooms to avoid possible discussion between patients and proxy respondents. The MSIS-29 is a self-report measurement which assesses the physical and psychological impact of MS on daily life. Both subscales consist of 20 items and 9 items, respectively. Scores on the individual items are added and transformed to a 0–100 scale, thereby generating two summary scores of both scales. Higher scores indicate worse health [2]. High values for test-retest reliability were found in the initial validation of the MSIS-29, which support use in longitudinal studies (intraclass correlation (ICC) of 0.94 and 0.87 for the physical scale and the psychological scale, respectively). A possible interpretation of MSIS-29 scores could be to categorise scores of 0–19 as 'no problems', 20–39 as 'few problems', 40–59 as 'moderate problems', 60–79 as 'quite a few problems' and 80–100 as 'extreme problems'[20]. For this study, the Dutch version of the MSIS-29 was used, which is an in-house translation of the original English version that was subsequently validated in a large study across eight European countries [21]. The partners completed a modified version of the MSIS-29 in which all items were phrased in the third person perspective. The proxy respondents were instructed to assess the patient as the proxy thought the patient would rate his or herself [22]. They had to complete the MSIS-29 keeping in mind the following question: 'How do you think the patient experiences the impact of MS on his/her life?' A psychometric evaluation of the MSIS-29, when completed by proxy respondents, was previously performed and the results were supportive towards using the MSIS-29 in a proxy sample [23]. Test re-test reliability showed an ICC of 0.87 for the physical scale and an ICC of 0.83 for the psychological scale. Additionally, data on disability, mood and cognition, were collected in the patient sample to investigate possible influence of these factors on mean differences between patients and proxy respondents. Cognitive performance was assessed by applying the Brief Repeatable Battery of Neuropsychological Tests (BRB-N) [5,24]. The BRB-N is a test battery consisting of five tests, each measuring a different area of cognitive functioning, including verbal learning and memory, visuospatial learning, attention and concentration, information processing and semantic verbal fluency. The Hospital Anxiety and Depression Scale (HADS) was used to assess the mood status of the patient. The HADS is a 14-item screening instrument which is used to screen for possible anxiety disorders and depression. It contains two 7-item scales: one for anxiety and one for depression both with a score range of 0–21 [25]. Both domains were used to define the mood status of the patient. A trained doctor assessed neurological impairment and disability with the Expanded Disability Status Scale (EDSS) [26]. MS subtype was available for all patients.

#### Follow-up

Two years after baseline assessment, patients and proxy respondents were contacted again and invited to participate in the follow-up study. According to the preference of the patient, the visit was scheduled at the MS Center or a trained medical student visited the patient and partner at home. During assessment, both patient and proxy respondent completed the MSIS-29 and data on mood status of the patient was collected by the HADS. The proxy respondent was asked to complete the MSIS-29 in a separate room of the house. Although the aim was that the partner was present during the follow-up visit, this was not always possible. When the partner was not present, the questionnaires were given along with the patient to take home or were left after the home visit with explicit instructions that they had to complete the questionnaires independently from the patient. Due to practical restrictions the BRB-N was not performed at follow-up. In the case of a home visit, the EDSS score was assessed by using an interview-based questionnaire. This interview-based questionnaire was developed for patients who are unable to continue visiting the outpatient clinic for follow-up measurement in longitudinal studies or clinical trials [27]. Additionally, global ratings of both patients and proxy respondents of the extent to which the patient stayed stable, improved or deteriorated over the past two years, were collected to validate possible changes on the MSIS-29. These ratings were collected by using a so-called 'transition question', which requires the subject to compare their prior health status to their current situation [28]. In this study, patients were asked: 'How do you feel, regarding to your MS, in comparison to two years ago?' Answer categories included the following items: 'a lot worse' – 'worse' – 'the same' – 'better' – 'a lot better'. The proxy respondent was also asked how they thought the patient felt in comparison to two years ago.

### Data analyses

#### Patient-proxy differences and agreement at baseline and follow-up

The data were initially analysed separately for baseline and follow-up for which several statistical strategies were used.

First, mean differences (mean patient scores minus mean proxy scores) between both groups were calculated for both scales to get insight in the direction and magnitude of these differences. Paired student's t-tests were used to examine whether these differences were statistically significant and would therefore provide evidence of systematic over- or underreporting by one group. Such systematic bias can occur when, for example, proxy respondents consistently report a lower or higher impact of MS than the patients themselves. In this case there can still be an excellent correlation between the two groups but a poor agreement. In line with other literature on proxy measurements, the effect size (*d*) was used to estimate the magnitude of the systematic differences [29]. The effect size can be estimated by standardising the mean differences to their standard deviations (mean directional difference/SD of difference). Since this method is similar to calculating effect sizes (*d*) used in paired t-tests it seems reasonable to apply the same classification: *d *= 0.20 indicates a small bias, *d *= 0.50 indicates a moderate bias, *d *= 0.8 indicates a large bias [29].

Patient-proxy agreement was also examined by calculating the correlation between ratings of patients and proxy respondents. An appropriate statistic is the intraclass correlation coefficient (ICC), which is calculated as the ratio of the variance between subjects (variance of interest) and the total variance [30]. For this study the two-way random model for absolute agreement was used [31]. Standards for interpreting ICC values are arbitrary but one can apply the standard reliability criteria of an ICC > 0.70, which is adequate and an ICC > 0.80 is preferred [32]. In addition, patient-proxy agreement was assessed by calculating the percentages of exact agreement and global agreement on responses between patient-partner pairs. Exact agreement is the percentage of patient responses identical to the responses of the proxy (e.g. both patient and proxy respondent score 2 on item 12). Global agreement refers to the percentage of agreement within one response category in both directions (e.g. patient scores 2 on item 12 and the proxy respondent scores 1, 2 or 3 on this item) [30].

#### Patient-proxy agreement on change over time

To evaluate patient-proxy agreement on change over time, mean change scores (follow-up minus baseline) on the MSIS-29 were calculated for both patients and proxy respondents. Subsequently, agreement on possible change over time was assessed by calculating ICCs between the mean change scores.

Next, the items of the transition questions were dichotomised into patients who deteriorated ('a lot worse' – 'worse') and those who did not ('the same' – 'better' – 'a lot better'). The latter group is in the remainder of this paper indicated as 'stable'. The same was done for proxy respondents who thought the patients deteriorated or stayed 'stable' over the last two years. A Kappa coefficient (κ) was calculated to assess a chance corrected agreement for the transition ratings of both groups.

Finally, both patient and proxy samples were divided according to the dichotomous transition question (deteriorated – stable). Again, change scores were calculated for both groups in order to see if the change scores on the MSIS-29 were in concordance with the transition ratings.

#### Factors affecting patient-proxy differences

Different variables that could have influenced on patient-proxy differences at baseline and follow-up were investigated. These variables included: disability, mood, cognition of the patient and gender of the proxy respondent. For each variable the sample was divided into different subgroups, according to variable-specific criteria. Subsequently, the mean directional differences between patients and proxy respondents were calculated for the corresponding MSIS-29 scores in each subgroup. One-way ANOVA analyses were performed to see if these subgroups differed significantly from each other.

The EDSS score of the patient was divided according to the following classification: 0.0–3.5; 4.0–6.0; 6.5–8.0. Possible influence of mood was investigated by dividing the sample into subgroups defined on the HADS score. The following criteria were used: ≤ 7 = no clinically levels of anxiety and depression; 8 – 10 = clinically borderline; ≥ 11 = clinically definite levels of anxiety and depression [33]. In order to see if cognitive impairment was of influence, the sample was divided into three subgroups: normal BRB-N scores, 1 or 2 abnormal BRB-N scores and 3 or more abnormal BRB-N scores. Cognitive impairment on one of the tests of the BRB-N was defined as two standard deviations below the mean reported for healthy subjects [5,24]. Although there is no universal consensus of how to define or diagnose cognitive impairment in MS, the frequently used criteria of three or more abnormal tests scores on the BRB-N was used to define cognitive impairment [34]. Finally, the sample was divided according to the gender of the proxy.

## Results

### Study sample

Follow-up data was available for 56 of the 59 patient-proxy couples. Reasons for 'lost to follow-up' were that two patients were reluctant to participate due to their busy schedules and one patient was withdrawn from the study by his partner due to severe cognitive impairment of the patient. The patients and proxy respondents had a mean follow-up duration of 750 days, which equals a time period of 2.06 years (range years: 1.8 – 2.3, SD = 36.2 days). All patients and proxies were living together, except for one couple. This couple lived together only during weekends; on the other days of the week the patient stayed in a rehabilitation center.

Table 1 lists the characteristics of the patients and proxy respondents, the mean total scores on the physical and psychological scale of the MSIS-29 and the number of patients in each subgroup of the EDSS, the HADS and the BRB-N at baseline and follow-up.

**Table 1:** Characteristics of patients and proxy respondents at baseline and follow-up

	**Baseline**		**Follow-up**	
	Patients	Proxy respondents	Patients	Proxy respondents

Total	59	59	56	56
Female (n)	41	19	40	17
Age (years)*	47.8 (8.9)	49.2 (9.0)	50.6 (9.1)	52.4 (9.9)
Years since MS onset*	12.8 (7.2)	-	14.8 (7.4)	-
Type of MS (n)				
Relapsing-remitting	28	-	26	-
Secondary progressive	19	-	18	-
Primary progressive	10	-	10	-
Other	2	-	2	-
MSIS-29				
Physical scale*	42.0 (24.1)	42.8 (25.8)	45.1 (25.3)	50.0 (25.8)
Psychological scale*	29.0 (22.6)	33.3 (22.5)	30.1 (23.5)	39.0 (24.4)
				
EDSS (median)	4.5	_	5.5	_
				
HADS (n, depression/anxiety)				
≤ 7	42/37	_	_	_
8–10	7/10	_	_	_
≥ 11	10/12	_	_	_
				
BRB-N (n)				
0 abnormal scores	46	_	_	_
1–2 abnormal scores	6	_	_	_
≥3 abnormal scores	5	_	_	_
Missing	2	_	_	_

*Values are mean (SD)

MSIS-29: Multiple Sclerosis Impact Scale, EDSS: Expanded Disability Status Scale, HADS: Hospital Anxiety and Depression Scale, BRB-N: Brief Repeatable Battery of Neuropsychological Tests

### Patient-proxy differences and agreement at baseline and follow-up

Table 2 shows the mean directional differences between patients and proxy respondents and values of agreement, at baseline and follow-up.

**Table 2:** Patient-proxy differences and agreement on baseline and follow-up

	**MSIS-29**	**Directional difference^a ^(Mean ± SD) (95% CI)**	***d***	**ICC (95% CI)**	**Exact agreement (%)**	**Global agreement (%)**
**Baseline **(n = 59)	Physical scale	-0.8 ± 15.3 (-4.9 – 3.2)	0.1	0.81 (0.70–0.88)	47.4	83.1
	Psychological scale	-4.4 ± 17.0 (-8.7 – 0.1)	0.3	0.72 (0.55–0.82)	44.6	82.5
**Follow-up **(n = 56)	Physical scale	-4.8 ± 12.7^b ^(-8.2 – 1.4)	0.4	0.86 (0.76–0.92)	43.3	82.6
	Psychological scale	-8.9 ± 18.8^b ^(-14.0 – 3.9)	0.5	0.65 (0.42–0.79)	38.1	81.0

^a ^Patient minus proxy respondent; negative scores indicate that the proxy scores higher than the patient.

^b ^Statistically significant difference between mean score of patient and proxy groups (P < 0.05)

MSIS-29: Multiple Sclerosis Impact Scale, SD: standard deviation, ICC: intraclass correlation coefficient, *d*: effect size, CI: confidence interval

At baseline, the mean directional difference on the physical score was minimal (-0.8 ± 15.3) and there was no evidence for systematic bias (*d *= 0.1). The mean directional difference for the psychological scale was -4.4 ± 17.0, but this difference was not statistically different. At follow-up, statistically significant mean differences were seen for the physical scale (-4.8 ± 12.7, p = 0.006) and the psychological scale (-8.9 ± 18.8, p = 0.001). However, both scales showed moderate systematic bias.

ICCs for the physical scale were larger than 0.80, at baseline and follow-up. At both time points, the ICC for the psychological scale was lower than the ICC for the physical scale: 0.72 at baseline and 0.65 at follow-up.

At baseline, there was an exact agreement of 47.4% on the physical scale and 83.1% of the patients and proxy respondents answered within one adjacent category. Exact and global agreement for the psychological scale was 44.6% and 82.5%, respectively. At follow-up, the average proportion of exact agreement on the physical scale was 43.3% and global agreement was 82.6%. Identical responses were seen for 38.1% of the patients and proxy respondents and 81.0% responded within one adjacent category.

### Patient-proxy agreement on change over time

Table 3 shows the mean change scores (follow-up minus baseline) for both scales, in both groups. Positive scores indicate a higher mean scale score on the MSIS-29 at follow-up. Change scores for both patients and proxy respondents were positive, indicating a higher score on the MSIS-29 at follow-up. Patients had a mean change score of 3.0 ± 13.5 on the physical scale. A small mean change score was seen on the psychological scale (1.0 ± 16.4). The change scores for the proxy respondents on the physical and the psychological scale were 7.1 ± 16.6 and 5.6 ± 22.5, respectively. The mean change scores of the proxy respondents on both scales appeared to be larger than the mean change scores of the patient sample. However, independent t-test showed that these differences were not statistically significant (p ≥ 0.05).

**Table 3:** Change scores on both scales of the MSIS-29

	**Δ MSIS-29 physical scale^a ^(mean ± SD)**	**Δ MSIS-29 psychological scale^a ^(mean ± SD)**
Patient	3.0 ± 13.5	1.0 ± 16.4
Proxy	7.1 ± 16.6	5.6 ± 22.5
ICC Δ MSIS-29 (95% CI)	0.30 (0.06–0.53)	0.42 (0.19–0.61)

^a ^Mean score at follow-up minus mean score at baseline

MSIS-29: Multiple Sclerosis Impact Scale, CI: confidence interval

ICCs between the mean change scores are displayed in the last line of table 3; poor agreement (ICC = 0.30; 95% CI: 0.06–0.53) was seen for the physical scale and fair agreement was seen for the psychological scale (ICC = 0.42; 95% CI: 0.19–0.61).

Data on the transition ratings showed that 39 patients judged their situation as deteriorated as opposed to 17 patients who indicated that they did not deteriorate in the past two years. In the proxy sample, also 39 proxies indicated that the patient deteriorated and 17 proxies indicated that the patients had not deteriorated. It should be emphasised that, although the distribution of transition ratings numbers was identical in both samples, they did not relate to the same patient-proxy couples. In fact, the strength of the agreement between the transition ratings was only moderate (κ = 0.58). Table 4 shows the mean change scores according to transition ratings of patients and proxy respondents. It can be observed from table 4 that, although the patients indicated that they stayed stable (or even improved) over the past two years, they did show a larger mean change score on both scales when compared to patients who indicated that they deteriorated over the past two years. The opposite is seen in the proxy sample: proxy respondents who indicated that the patients stayed stable (or improved) over the past two years had smaller change score on the MSIS-29 when compared to proxies who indicated that patients had deteriorated.

**Table 4:** mean changes scores on both scales of the MSIS-29 according to transition ratings

**Patient**		
**Transition rating**	**Δ MSIS-29 physical scale^a ^(mean ± SD)**	**Δ MSIS-29 psychological scale^a ^(mean ± SD)**

Stable^b ^(n = 17)	4.3 ± 12.8	1.8 ± 19.2
Deteriorated^c ^(n = 39)	2.4 ± 14.0	0.6 ± 15.2
**Proxy**		
**Transition rating**	**Δ MSIS-29 physical scale^a ^(mean ± SD)**	**Δ MSIS-29 psychological scale^a ^(mean ± SD)**
Stable^b ^(n = 17)	5.4 ± 13.5	2.0 ± 18.6
Deteriorated^c ^(n = 39)	7.8 ± 17.9	7.1 ± 24.1

^a ^Mean score at follow-up minus mean score at baseline

^b ^combines the transition ratings 'the same' – 'better' – 'a lot better', indicating both stability and improvement

^c ^combines the transition ratings 'worse' and 'a lot worse'.

MSIS-29: Multiple Sclerosis Impact Scale

### Factors affecting patient-proxy differences

At baseline and follow-up, one-way ANOVA analyses showed no significant differences between the three subgroups of the EDSS and the HADS. There were also no significant differences between the subgroups of the BRB-N scores at baseline.

Moderate systematic bias was seen on the psychological scale of the MSIS-29 for female proxy respondents, both at baseline (-8.37 ± 17.1, p = 0.047, *d *= 0.5) and follow-up (-10.8 ± 17.5, p = 0.022, *d *= 0.6). There was no gender bias seen on the physical scale.

## Discussion

The aim of this study was two-fold; to examine patient-proxy agreement on the impact of MS on daily life at different points in time and to get more insight in patient-proxy agreement on change over time.

The positive mean differences that were found at baseline and follow-up indicated a tendency for proxy respondents to report more disease impact of MS than the patient did themselves. This tendency of reporting more symptoms and lower levels of functioning by proxy respondents compared to the patients themselves, is in line with other studies on this topic [10,35]. In contrast to some of the other longitudinal proxy studies [13,14,19] we did not see an improvement in agreement over time. Mean directional differences at follow-up were actually statistically significant for both the physical and the psychological scale, which points towards systematic overestimation by proxy respondents. Effect sizes showed that the magnitude of this bias was moderate for both scales.

At both time points, the ICC showed good agreement for the physical scale and a slightly lower but still adequate agreement for the psychological scale. Both ICCs and the percentages for exact and global response agreement were lower for the psychological scale than for the physical scale, indicating a lower agreement for the psychological scale. This supports the current view in patient-proxy agreement studies that better agreement is demonstrated when it comes to the more objective, more observable questions but less agreement is seen when it comes to the more subjective, less observable questions [10,11].

With respect to change over time, proxy respondents appeared to report a larger change than the patients did themselves, but these differences were not significant. On the other hand, ICCs between the change scores on the MSIS-29 were poor, indicating a low level of patient-proxy agreement on the change scores. However, since an ICC is based on the variance of the sample, a lack of variance in change scores could also have caused the low ICCs, rather than lack of patient-proxy agreement [36]. Moreover, part of the patient sample was rather stable over the study period. Especially in these cases, differences between the two measurements are mainly caused by measurement error or random error, which will lower the calculated ICCs. Other studies showed contrasting results ranging from poor agreement [14,16,17] to moderate agreement on change over time [19,37].

When the change scores were validated against the transition ratings, a remarkable finding was observed; while patients indicated that they stayed stable over the preceding two years, they showed a larger increase on the MSIS-29 when compared to patients who indicated that they had deteriorated over that time. These patients showed a lower increase on the MSIS-29. When the same comparison was made for the proxy respondents it was seen that the transition ratings of the proxy respondents were in concordance with the mean change scores. These results could suggest that the ability to rate possible changes over time might be better for proxy respondents. Although the loss to follow-up was limited in this study, the effect of non-random drop out is hard to exclude.

Besides this, possible methodological issues concerning transition ratings could compromise results [28]. Research has shown that it appears to be very difficult for the patient to compare their initial health state to their current health status and that their judgement of change is actually based on their current health status. It is not known whether the change scores or the transition ratings are more accurate and if they actually measure the same concept [38]. The differences between the transition ratings and the change scores could also reflect measurement error or may be due to different perceptions of the meaning of a change [38]. Also, the patients in this sample stayed relatively stable over the two years. The change scores that were measured could therefore have consisted mostly of measurement error instead of actual change in disease impact. Moreover, in a longitudinal setting, one should also be aware of the possible occurrence of response shift which may influence the results.

Several other factors that could possibly have influenced the mean directional differences, and could therefore have caused systematic bias, were examined. Disability and mood status (anxiety and depression) did not seem to significantly influence the differences between patients and proxy respondents at baseline and follow-up. Cognitive performance did not have an effect at baseline. The finding that disability, cognitive functioning and mood did not seem to have an influence should be interpreted with caution, since these findings are based on a small sample. This could have resulted in a low power and therefore false negative findings (Type II error). Future research focusing on proxy measurements in MS should therefore be performed in larger and/or more cognitively impaired samples. Unfortunately, there were no data on cognitive performance at follow-up so it is unclear whether there was an effect. In contrast to male proxy respondents, female proxy respondents seemed to consistently overestimate the psychological impact of MS at baseline and follow-up. There was no gender bias found for the physical scale. However, there is no consensus on influence of gender on patient-proxy differences and this finding should therefore be considered with caution. Another limitation of this study was the use of partners as proxies. Whether these results are also applicable on other proxies, such as healthcare providers, also remains to be investigated.

## Conclusion

Findings from this study show acceptable levels of patient-proxy agreement both at baseline and follow-up, especially on the physical scale. Proxy respondents could therefore play a supportive role in cross-sectional studies by providing valuable information in situations when the patient is not capable due to limitations that interfere with reliable self-assessment. The level of patient-proxy agreement on change of disease impact appeared to be low. The finding that proxy respondents were better assessors of change over time is striking, but should be interpreted by taking into account the limitations of this study. Nonetheless, the value of proxy respondents in MS research remains an important issue and further research into the validity and reliability of proxy respondents in longitudinal studies is needed.

## Competing interests

The author(s) declare that they have no competing interests.

## Authors' contributions

FAHL participated in the data collection and coordination, performed the statistical analysis together with BMJU and drafted the manuscript. JJK contributed to the data collection and drafting of the manuscript. MB participated in the data collection and coordination. BMJU conceived of the study concept and participated in its design and coordination and drafting of the manuscript together with MK, AJT, HMP, and CHP. All authors read and approved the final manuscript.

## Pre-publication history

The pre-publication history for this paper can be accessed here:



## Supplementary Material

Additional file 1 Multiple Sclerosis Impact Scale (MSIS-29). The MSIS-29 is the questionnaire that was used in this study. Click here for file
