# Supplementary material for: Longitudinal proxy measurements in multiple sclerosis: patient-proxy agreement on the impact of MS on daily life over a period of two years
Source: BMC Neurol. 2008 Feb 28;8:2. doi: 10.1186/1471-2377-8-2 (PMC2270863; doi:10.1186/1471-2377-8-2)
Supplement: Additional file 1 — Multiple Sclerosis Impact Scale (MSIS-29). The MSIS-29 is the questionnaire that was used in this study. [file 1471-2377-8-2-S1.doc]

Multiple Sclerosis Impact Scale (MSIS-29)

- The following questions ask for your views about the impact of MS on your day-to-day life **during the past two weeks**
- For each statement, please **circle** the **one** number that **best** describes your situation
- Please answer **all** questions

| | **In the past two weeks, how much has your MS limited your ability to...** | | Not at all | A little | Moderately | Quite a bit | Extremely | | --- | --- | --- | --- | --- | --- | --- | |  | | | | | | | | **1.** | **Do physically demanding tasks?** | 1 | 2 | 3 | 4 | 5 | | **2.** | **Grip things tightly (e.g. turning on taps)?** | 1 | 2 | 3 | 4 | 5 | | **3.** | **Carry things?** | 1 | 2 | 3 | 4 | 5 | |
| --- | --- | --- | --- | --- | --- | --- | --- | --- | --- | --- | --- | --- | --- | --- | --- | --- | --- | --- | --- | --- | --- | --- | --- | --- | --- | --- | --- | --- | --- | --- | --- | --- | --- | --- | --- |

| | **In the past two weeks, how much have you been bothered by...** | | Not at all | A little | Moderately | Quite a bit | Extremely | | --- | --- | --- | --- | --- | --- | --- | |  | | | | | | | | **4.** | **Problems with your balance?** | 1 | 2 | 3 | 4 | 5 | | **5.** | **Difficulties moving about indoors?** | 1 | 2 | 3 | 4 | 5 | | **6.** | **Being clumsy?** | 1 | 2 | 3 | 4 | 5 | | **7.** | **Stiffness?** | 1 | 2 | 3 | 4 | 5 | | **8.** | **Heavy arms and/or legs?** | 1 | 2 | 3 | 4 | 5 | | **9.** | **Tremor of your arms or legs?** | 1 | 2 | 3 | 4 | 5 | | **10.** | **Spasms in your limbs?** | 1 | 2 | 3 | 4 | 5 | | **11.** | **Your body not doing what you want it to do?** | 1 | 2 | 3 | 4 | 5 | | **12.** | **Having to depend on others to do things for you?** | 1 | 2 | 3 | 4 | 5 | |  | | | | | | | | **Please check that you have answered all the questions before going on to the next page** | | | | | | | | **®2000 Neurological Outcome Measures Unit, 4^th^ Floor Queen Mary Wing, NHNN, Queen Square, London WC1N 3BG, UK** | | | | | | | |
| --- | --- | --- | --- | --- | --- | --- | --- | --- | --- | --- | --- | --- | --- | --- | --- | --- | --- | --- | --- | --- | --- | --- | --- | --- | --- | --- | --- | --- | --- | --- | --- | --- | --- | --- | --- | --- | --- | --- | --- | --- | --- | --- | --- | --- | --- | --- | --- | --- | --- | --- | --- | --- | --- | --- | --- | --- | --- | --- | --- | --- | --- | --- | --- | --- | --- | --- | --- | --- | --- | --- | --- | --- | --- | --- | --- | --- | --- | --- | --- | --- | --- | --- | --- | --- | --- | --- | --- | --- | --- | --- | --- | --- | --- | --- | --- | --- | --- | --- |

| | **In the past two weeks, how much have you been bothered by...** | | Not at all | A little | Moderately | Quite a bit | Extremely | | --- | --- | --- | --- | --- | --- | --- | |  | | | | | | | | **13.** | **Limitations in your social and leisure activities at home?** | 1 | 2 | 3 | 4 | 5 | | **14.** | **Being stuck at home more than you would like to be?** | 1 | 2 | 3 | 4 | 5 | | **15.** | **Difficulties using your hands in everyday tasks?** | 1 | 2 | 3 | 4 | 5 | | **16.** | **Having to cut down the amount of time you spent on work or other daily activities?** | 1 | 2 | 3 | 4 | 5 | | **17.** | **Problems using transport (e.g. car, bus, train, taxi, etc.)?** | 1 | 2 | 3 | 4 | 5 | | **18.** | **Taking longer to do things?** | 1 | 2 | 3 | 4 | 5 | | **19.** | **Difficulty doing things spontaneously (e.g. going out on the spur of the moment)?** | 1 | 2 | 3 | 4 | 5 | | **20.** | **Needing to go to the toilet urgently?** | 1 | 2 | 3 | 4 | 5 | | **21.** | **Feeling unwell?** | 1 | 2 | 3 | 4 | 5 | | **22.** | **Problems sleeping?** | 1 | 2 | 3 | 4 | 5 | | **23.** | **Feeling mentally fatigued?** | 1 | 2 | 3 | 4 | 5 | | **24.** | **Worries related to your MS?** | 1 | 2 | 3 | 4 | 5 | | **25.** | **Feeling anxious or tense?** | 1 | 2 | 3 | 4 | 5 | | **26.** | **Feeling irritable, impatient, or short tempered?** | 1 | 2 | 3 | 4 | 5 | | **27.** | **Problems concentrating?** | 1 | 2 | 3 | 4 | 5 | | **28** | **Lack of confidence?** | 1 | 2 | 3 | 4 | 5 | | **29.** | **Feeling depressed?** | 1 | 2 | 3 | 4 | 5 | |  | | | | | | | | **Please check that you have circled ONE number for EACH question** | | | | | | | | **© 2000 Neurological Outcome Measures Unit, 4^th^ Floor Queen Mary Wing, NHNN, Queen Square, London WC1N 3BG, UK** | | | | | | | |
| --- | --- | --- | --- | --- | --- | --- | --- | --- | --- | --- | --- | --- | --- | --- | --- | --- | --- | --- | --- | --- | --- | --- | --- | --- | --- | --- | --- | --- | --- | --- | --- | --- | --- | --- | --- | --- | --- | --- | --- | --- | --- | --- | --- | --- | --- | --- | --- | --- | --- | --- | --- | --- | --- | --- | --- | --- | --- | --- | --- | --- | --- | --- | --- | --- | --- | --- | --- | --- | --- | --- | --- | --- | --- | --- | --- | --- | --- | --- | --- | --- | --- | --- | --- | --- | --- | --- | --- | --- | --- | --- | --- | --- | --- | --- | --- | --- | --- | --- | --- | --- | --- | --- | --- | --- | --- | --- | --- | --- | --- | --- | --- | --- | --- | --- | --- | --- | --- | --- | --- | --- | --- | --- | --- | --- | --- | --- | --- | --- | --- | --- | --- | --- | --- | --- | --- | --- | --- | --- | --- | --- | --- | --- | --- | --- | --- | --- | --- | --- | --- | --- | --- | --- | --- | --- |
